# Modeling Light-Induced Chromophore Hydration in the Reversibly Photoswitchable Fluorescent Protein Dreiklang

**DOI:** 10.3390/molecules28020505

**Published:** 2023-01-04

**Authors:** Bella L. Grigorenko, Igor V. Polyakov, Alexander V. Nemukhin

**Affiliations:** 1 Department of Chemistry, M.V. Lomonosov Moscow State University, Moscow 119991, Russia; 2 N.M. Emanuel Institute of Biochemical Physics, Russian Academy of Sciences, Moscow 119334, Russia

**Keywords:** fluorescent proteins, photoswitching, protein Dreiklang, hydration reaction, molecular modeling, excited-state properties, conical intersection points, quantum chemistry, QM/MM

## Abstract

We report the results of a computational study of the mechanism of the light-induced chemical reaction of chromophore hydration in the fluorescent protein Dreiklang, responsible for its switching from the fluorescent ON-state to the dark OFF-state. We explore the relief of the charge-transfer excited-state potential energy surface in the ON-state to locate minimum energy conical intersection points with the ground-state energy surface. Simulations of the further evolution of model systems allow us to characterize the ground-state reaction intermediate tentatively suggested in the femtosecond studies of the light-induced dynamics in Dreiklang and finally to arrive at the reaction product. The obtained results clarify the details of the photoswitching mechanism in Dreiklang, which is governed by the chemical modification of its chromophore.

## 1. Introduction

Fluorescent proteins, which can switch between bright and dark states for multiple photocycles, are important markers for bioimaging. The engineered protein Dreiklang [1], a member of the green fluorescent protein (GFP) family with a chromophore formed from the Gly65-Tyr66-Gly67 tripeptide, is a notable moiety, because its optical properties are modified in the chemical reaction of its chromophore. Mostly, the photoswitching in fluorescent proteins is due to the conformational changes of the chromophore, or to the tuning of the hydrogen bond network near the chromophore [2,3,4,5,6,7,8,9,10,11]. As proposed in the pioneering work of Brakemann et al. [1], a structural water molecule in Dreiklang attaches to its chromophore at the imidazolinone ring upon light illumination at 405 nm (3.06 eV). This process converts the protein from the fluorescent (ON) state to the non-fluorescent (OFF) state. The ON-state can be restored by the light illumination of the OFF-state at 365 nm (3.40 eV), or the recovery reaction can take place spontaneously in the dark. Ref. [1] describes the synthesis, steady-state absorption spectra, electrospray ionization mass spectra and crystal structures of the protein in the ON- and OFF-states.

**Figure 1:**
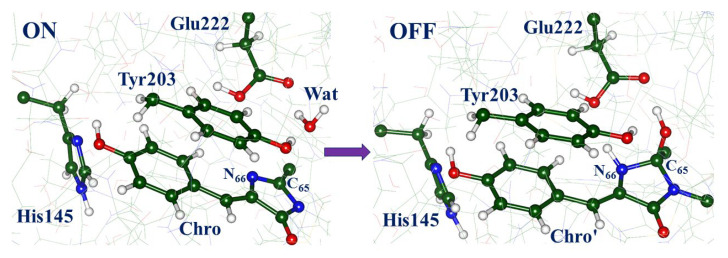
Fragments of protein structures corresponding to the ON- (left) and OFF- (right) states in Dreiklang, illustrating the reactants and the products of the hydration reaction. In this and in other figures, carbon atoms are shown in green, oxygen in red, nitrogen in blue, hydrogen in white.

Tentative mechanisms of chemical reactions of the chromophore hydration/dehydration in Dreiklang are proposed in the subsequent works [12,13,14]. To clarify these approaches, we show in Figure 1 molecular models of the reactants and the products in the ON → OFF reaction in Dreiklang, drawn by motifs of the computationally derived protein structures described in Ref. [13]. The paper [13] reports the protein models obtained in quantum mechanics/molecular mechanics (QM/MM) calculations, which are well consistent with the crystal structures [1] deposited to the Protein Data Bank [15] as PDB ID 3ST4 (ON-state) and PDB ID 3ST3 (OFF-state). Ref. [13] shows that these models allow one to compute the energy profile for the OFF → ON reaction of thermal recovery of the fluorescent state, which is also well consistent with the experimental data [1]. The panels in Figure 1 illustrate fragments of the chromophore-containing pocket with the initial chromophore Chro and the reactive water molecule Wat (left side) and the hydrated chromophore Chro’ (right side). In the simplest representation, the water molecule Wat splits into the hydroxyl ion and the proton, which are attached to the C_65_ and N_66_ atoms of Chro, respectively. The detailed mechanism of this photochemical reaction should take into consideration environmental molecular groups; the panels in Figure 1 show the nearest to the chromophore amino acid residues, His145, Tyr203, Glu222, which are important in the hydration reaction, according to the proposals formulated in Refs. [12,13,14].

Lacombat et al. [12] described the results of femtosecond spectroscopy studies of Dreiklang and tentatively suggested the mechanism of the light-induced hydration reaction after the illumination of the protein in the ON-state. The authors presumed that the S_0_ → S_1_ local excitation of the *cis*-neutral chromophore leads to the evolution of the system on the excited-state potential energy surface (PES), followed by the switch to the plausible ground-state reaction intermediate called X, and later to the OFF-state with the hydrated chromophore. The X intermediate, which, by the authors’ suggestion, is an adduct of the anionic chromophore with the water molecule, is characterized by the transient absorption band at 450 nm (2.76 eV) observed in the femtosecond experiments. The mechanistic scheme [12] assumes the excited-state electron and proton transfer with the participation of the nearby residues His145 and Glu222. The process includes chromophore protonation at N_66_ by the Glu222 side chain, followed by the addition of the hydroxyl at C_65_ (see Figure 1). For the reader’s convenience, we present an illustration of this proposal in the Supporting Materials for the present paper (Figure S1).

Sen et al. [14] suggested another mechanism of chromophore hydration following a systematical computational study of the nature of the excited states in Dreiklang. The authors applied several quantum chemistry and QM/MM approaches to estimate absorption spectra in Dreiklang and showed that, in the ON-state model systems with the neutral form of the chromophore and the neutral forms of His145 and Glu222, the levels of the locally excited S_1_ and the charge transfer (CT) state are nearly isoenergetic. The light-driven chromophore in a locally excited state S_1_ tends to relax into the CT state due to electron transfer from the neighboring residue Tyr203 to the chromophore (see Figure 1). To investigate possible excited-state pathways, QM/MM geometry optimization and short ab initio type molecular dynamics (AIMD) simulations based on the time-dependent density functional theory (TD DFT) approach were carried out. The results allowed the authors to propose a series of alternative steps in the mechanism as compared to Ref. [12], assuming involvement of the Tyr203 and Glu222 side chains, as illustrated in the Supporting Materials for the present paper (Figure S2). In particular, a few candidates for the critical reaction intermediate X were suggested in Ref. [14], promising a more elaborate characterization “in future studies”.

The primary goal of the present work was to model the pathways of the light-induced hydration reaction in Dreiklang by using complementary simulation tools as compared to Ref. [14]. We explored the relief of the CT excited-state PES with the state-averaged complete active space self-consistent field (CASSCF) method and located the minimum energy conical intersection (MECI) points of the charge transfer and the ground-state potential surface. Analysis of the further evolution of the system in the ground state by the QM/MM method allowed us to find the route to the OFF-state structure via the firmly established reaction intermediate X, thus providing a full characterization of the mechanism of the hydration reaction in Dreiklang. We also explore alternative protonation states of the amino acid residues next to the chromophore and discuss the role of proton wires in Dreiklang. The obtained results may be helpful for the consideration of other photoactive proteins with presumably hydrated GFP-like chromophores [16,17].

## 2. Results

**Figure 2:**
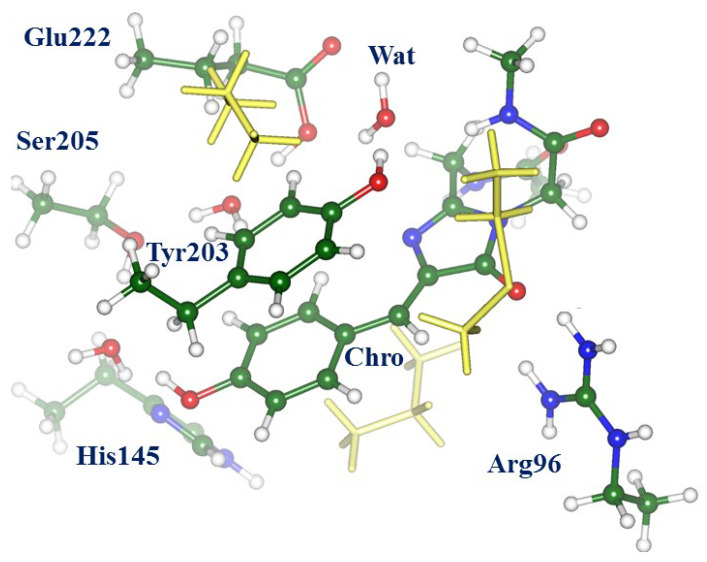
Composition of the molecular cluster mimicking the chromophore-containing pocket in Dreiklang in the configuration of the ON-state. Colored balls and sticks distinguish molecular groups, which are essential for the mechanism of the light-induced chromophore hydration reaction. Yellow sticks indicate molecular groups besides the key reaction participants, which are included in the system to surround atoms in the active site.

The geometry parameters and energies in the ground electronic state cited below were obtained in the QM/MM calculations with the NWChem software package [18] with the density functional theory M06-L functional [19] for QM and the AMBER99 force field parameters [20] for MM. Scans on the excited-state PES, search of the conical intersection points carried out with the Firefly package [21], and calculations of the vertical excitation energies and oscillator strengths carried out with the extended multiconfigurational quasi-degenerate perturbation theory in the second order (XMCQDPT2) [22] were performed for a large molecular cluster (see Figure 2), which was almost identical to the QM subsystem in QM/MM simulations in the full-protein model.

### 2.1. Structures and Spectra in the ON-State

**Figure 3:**
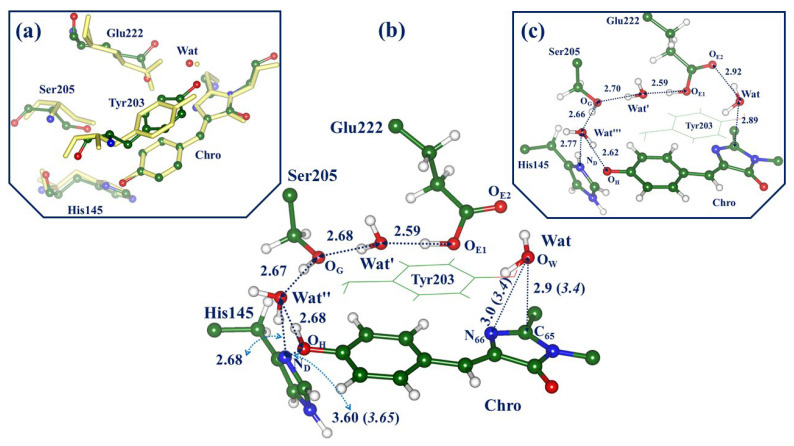
Model systems for the ON-state. (**a**) Superposition of fragments of the active site in the QM/MM-optimized structure (colored balls and sticks) and in the crystal structure PDB ID 3ST4 (yellow stick). Hydrogen atoms are not shown. (**b**) The QM/MM-optimized active site with the neutral form of Chro. (**c**) The QM/MM-optimized active site with the anionic form of Chro. The interatomic distances as given in Å. If two values are shown, the first value corresponds to the computed structure, and the value in parentheses (in italics) refers to the crystallography data.

The panels in Figure 3 illustrate the structural features of the ON-state models in the ground electronic state S_0_. Panel (a) shows a superposition of the key fragments of the active site from the QM/MM-optimized structure (colored balls and sticks) and those from the crystal structure PDB ID 3ST4 [1] (yellow stick). The central panel (b) illustrates the QM/MM-optimized active site with the neutral form of Chro, whereas panel (c) shows this site with the anionic Chro. The QM/MM energy of the model system with the neutral Chro is only1 kcal/mol lower than the energy of the system with the anionic Chro. According to the experimental absorption spectra of the protein in the ON-state [1,12], both protein conformations (with the neutral and anionic Chro) were observed in the experiments.

We comment that the geometry parameters optimized for the full-protein model at the QM/MM level are well consistent with the crystal structure PDB ID 3ST4 assigned to the ON-state [1]. In particular, this refers to the almost coplanar arrangement of the rings of the phenolic part of Chro (P-ring) and of the Tyr203 side chain, to the distance between Chro and His145, and even to the distances from the reactive water molecule Wat to Chro and Tyr203. Configurations of the Chro-Wat-Tyr203 subsystem are of particular importance for modeling.

Panels (b) and (c) show the important features common to the GFP-like proteins, namely the proton wires connecting the groups around Chro. Here, we distinguish the following proton translocation pathways: wire-1, connecting the Glu222 and His145 side chains (from O_ε1_(Glu) to N_δ_(His) via Ser205 and water molecules Wat’ and Wat”); wire-2, connecting Glu222 and Chro (from O_ε1_(Glu) to O_H_(Chro) via Ser205 and water molecules Wat’ and Wat”); wire-3, connecting Chro and His145 (from OH(Chro) to N_δ_(His) via Wat”). Wire-1 connects two possible protonation forms in the active site of the protein, neutral His145 and Glu222, and positively charged doubly protonated His145 and negatively charged deprotonated Glu222. Both forms can contribute to protein conformations; however, the analysis carried out in Ref. [14] favors the conformation with the neutral His145 and Glu222, as depicted in Figure 3b. Another short wire-3 accounts for the formation of the anionic Chro; this occurs if the proton initially bound to the O_H_ atom in the neutral form of Chro transfers to the N_δ_ atom in His145. If this process takes place, and Wat” re-orients, wire-2 operates as a conventional proton translocation pathway in the GFP-like proteins [23] connecting Chro and Glu (panel (c)).

Calculations of vertical excitation energies at the geometry configuration of the ON-state with the neutral chromophore (see Figure 3b) at the XMCQDPT2 level result in the following values: 2.80 eV (442 nm, 0.30 oscillator strength (o.s.)) for the locally excited state transition S_0_ → S_1_, and 3.00 eV (413 nm, 0.03 o.s.) for the charge transfer transition S_0_ → CT. The computed transition S_0_ → S_1_ for the ON-state conformation with the anionic chromophore (see Figure 3c) corresponds to 2.46 eV (503 nm, 0.78 o.s.). These values for the S_0_ → S_1_ excitation are well consistent with the steady-state absorption spectra for the ON-A protein form with the neutral Chro (411–413 nm) and the ON-B form with the anionic Chro (511 nm, higher intensity) [1,12]. We conclude that the models for the ON-state are a good starting point for the consideration of the photo-induced hydration reaction.

### 2.2. Evolution of the System in the Charge Transfer Excited State

**Figure 4:**
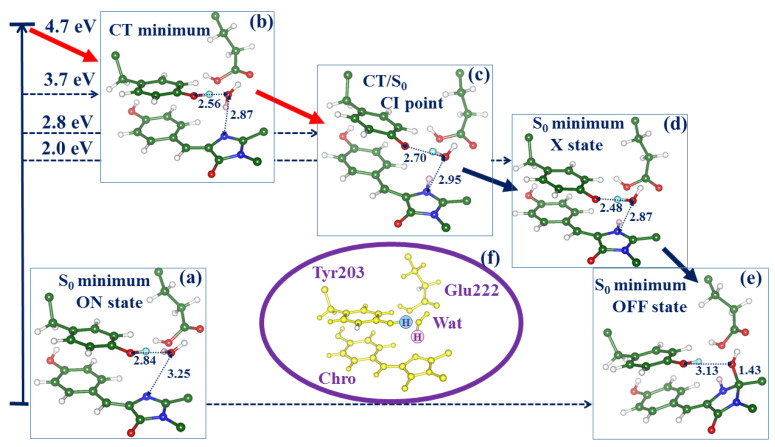
Mechanism of the ON–OFF light-induced reaction in the chromophore-containing pocket in Dreiklang by the results of the SA2-CASSCF calculations. Panels (**a**–**e**) show the chromophore, the side chains of Tyr203 and Glu222 and the water molecule Wat along the reaction pathway. The shuttling protons from Tyr203 and Wat are distinguished by cyan and pink colors, as clarified in panel (**f**). The interatomic distances are given in Å.

We distinguish the three lowest singlet-state energy levels at the structure of the ON-state with the neutral Chro (Figure 3b), denoted S_0_, S_1_ (local excited state), CT (charge transfer state). As shown in Ref. [14], the levels of the S_1_ and CT states at this geometry are almost isoenergetic, and their precise location depends on the applied theoretical level. Within the CASSCF framework, the first two roots refer to the S_0_ and CT states, whereas the S_1_ state appears as the third root. Therefore, the use of a practical state-average approach SA2-CASSCF allows us to explore the relief of the potential energy surface in the CT state, which finally leads to the hydration reaction pathway, as illustrated in Figure 4. In the panels in Figure 4, we show only four molecular groups, Chro, Wat, Tyr203, and Glu222. For better visibility, the shuttling protons, which initially reside on Tyr203 and Wat, are distinguished by the cyan and pink colors.

Minimization for the SA2-CASSCF second root initiated from the Frank–Condon point arrived at the structure shown in Figure 4b. In this computational scheme, the CT minimum lies approximately 1 eV lower than the S_0_ → CT excitation level. In comparison with the structure at the S_0_ minimum (Figure 4a), the reactant water molecule Wat stays notably closer to the tyrosine species and the imidazolinone ring (I-ring) of the chromophore. The subsequent evolution of the CT excited-state system is associated with the proton transfer along the chain Tyr-Wat-Chro (see Figure 4c). Estimates of the potential barrier from the located CT minimum give a value not exceeding 2 kcal/mol. The gradual descent of the CT potential energy after leaving the minimum energy area approaches the minimum energy conical intersection point (see Figure 4c). The switch to the ground state allowed us to locate the structure of the intermediate suspected in Ref. [12] as the ground-state photoproduct called “X”, and, finally, the structure of the reaction product (S_0_ minimum, OFF-state).

### 2.3. Back to the Ground State: The X and OFF Structures

**Figure 5:**
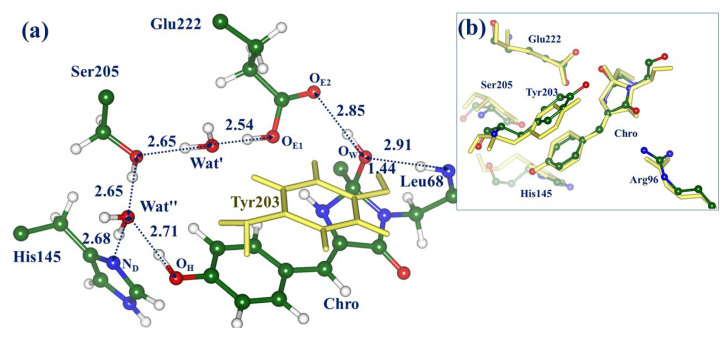
The active site in the QM/MM computationally derived OFF-state (panel (**a**)). Panel (**b**) shows superposition of fragments of the active site in the QM/MM-optimized structure (colored balls and sticks) and in the crystal structure PDB ID 3ST3 [1] (yellow stick). The interatomic distances are given in Å.

To complete the reaction, we return to the full-protein model and consider the structures X (the reaction intermediate) and OFF (the reaction product) at the ground-state PES. To this goal, the coordinates obtained at the SA2-CASSCF level (see panels (d) and (e) in Figure 4) were incorporated into the protein model and re-optimized at the QM/MM level. Figure 5 illustrates the structure in the OFF-state. Panel (a) shows the active site; the modified chromophore contains the corrupted I-ring with the covalently bound hydroxyl group; the latter is hydrogen-bound to the Glu222 side chain and the main chain fragment of Leu68. Panel (b) shows the superposition of fragments of the active site in the QM/MM-optimized structure (colored balls and sticks) and in the crystal structure PDB ID 3ST3 [1] (yellow stick). It is important to note that we did not use the coordinates of the experimental structure PDB ID 3ST3 as input data for simulations. Instead, we arrived at this construct in the course of simulated chemical transformations initiated from the ON-state structure. The agreement between the simulation results and experimental data for the OFF-state, which is of similar quality as for the starting ON-state, provides support to our simulations. The excitation energy computed at the located point at the XMCQDPT2 level (3.57 eV, 347 nm) also well agrees with the experimental absorption band maximum (3.65 eV, 340 nm) [1].

**Figure 6:**
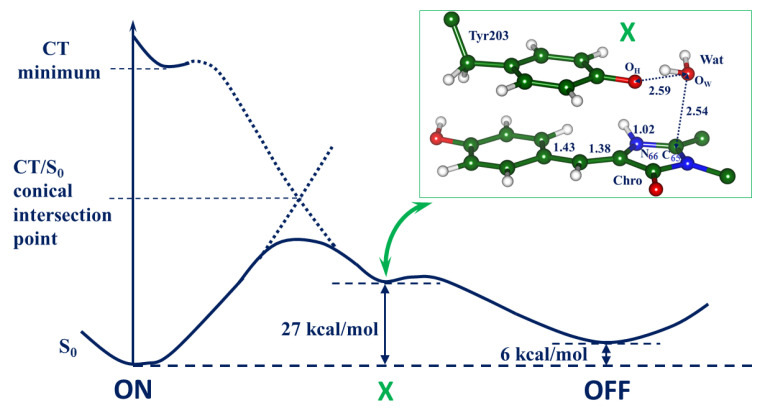
The summary energy diagram and the structure of the ground-state reaction intermediate X (inset). Energies of the ground-state structures ON, X, and OFF are computed at the QM/MM level. The interatomic distances are given in Å. Energies of the ground-state structures ON, X, and OFF are computed at the QM/MM level.

The main part in Figure 6 shows the computed energy diagram for the ON → OFF reaction with the energy values at the ground-state structures ON, X, and OFF computed at the QM/MM level. The most important fragment of structure X optimized in the full-protein model is shown in the inset in Figure 6. The reactant water molecule Wat stays close to the target I-ring; it is ready for the final elementary step, i.e., an attack of the hydroxyl ion from Wat on the C_65_ atom in the I-ring coupled with the proton transfer to Tyr203. Although we keep the same notation X for the ground-state reaction intermediate as suggested in Ref. [12], its nature differs from the initial proposal. Lacombat et al. [12] speculated that X might be an adduct of the anionic Chro with the water molecule. The results of our simulations suggest that X can be described as an adduct of the water molecule with the cationic Chro and anionic Tyr203 complex. According to the present QM/MM calculations, the energy of the X intermediate is 27 kcal/mol above the level of the initial ON-state structure. We find that the barrier separating the X and OFF structures is low, not exceeding 1 kcal/mol. The level of the OFF-state structure is 6 kcal/mol above the ON-state level, consistently with the observed [1] process of the thermal recovery of the fluorescent state in Dreiklang, as well as with the results of previous simulations [13]. The computed excitation energy at the X point, 2.79 eV (444 nm), agrees well with the observed transient absorption band maximum at 450 nm reported in the femtosecond spectroscopy study [12].

Table 1 summarizes the results for the absorption band maxima at the stationary points on the ground-state PES obtained in computations using the XMCQDPT2 method. Comparison with the experimental data [1,12] provides strong support to the simulations.

We conclude that the present simulations are consistent with the structural [1] and spectral [1,12] experimental observations, thus validating the suggested mechanism of the light-induced reaction in Dreiklang.

### 2.4. Other Protonation States of Amino Acid Residues in the Active Site

At the end, we comment on the importance of proton wires for biophysical properties of GFP-like proteins [24,25,26]. Here, we draw attention to the wires shown in Figure 3 and Figure 5. Besides the already mentioned wires connecting structures with the neutral and ionic forms of Chro, the wires connect the structures with the protonated and deprotonated forms of Glu222. We discussed above only the structures with the protonated side chain of Glu222 in the initial ON-state of the protein. Although this protonation state of Glu222 is supported by previous works [12,13,14], we cannot exclude the occurrence of the deprotonated Glu222 in some protein conformations. Wire-1, shown in Figure 3b, may serve as a low-barrier proton translocation pathway from O_ε1_(Glu222) to N_δ_(His145).

**Figure 7:**
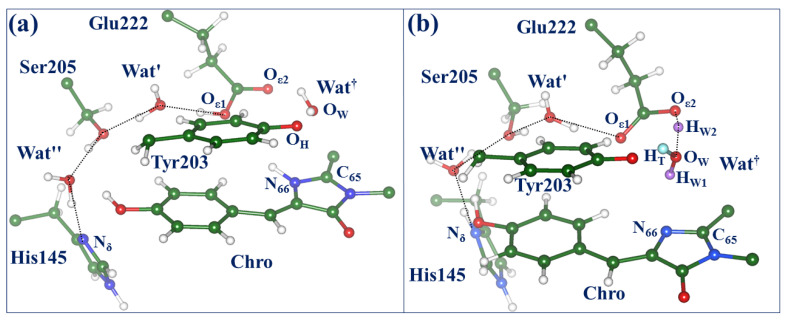
Structures of two possible conical intersection points CT/S_0_ on the ON → OFF photo-induced pathways. (**a**) The case of the neutral His145 and neutral Glu222 (see also Figure 4). (**b**) The case of the protonated His145 and initially deprotonated Glu222. In both cases, proton wires connecting atoms O_ε1_(Glu222) and N_δ_(His145) are shown by dotted lines. Symbol Wat† refers to the transient triatomic species formed from the reactive water molecule Wat and the proton from Tyr203.

Considering the latter scenario, we repeated SA-CASSCF calculations similar to those illustrated in Figure 4 but starting from the structure with the deprotonated (negatively charged) Glu222 and doubly protonated (positively charged) His145. In this case, we also were able to locate the conical intersection point CT/S_0_. Figure 7 compares the structures of two possible conical intersection points CT/S_0_ on the ON → OFF photo-induced pathways. The structure shown in Figure 7a corresponds to the previously discussed pathway (see Figure 4). The route via the CT/S_0_ conical intersection point shown in Figure 7b may be a more complicated issue. As before, a transient water species denoted as Wat^†^ formed from the reactive water molecule (with protons colored pink) and the proton (colored cyan) from Tyr203 occurs near the CT/S_0_ point. It should be noted that the excited-state energy relief from the CT minimum to the CT/S_0_ point is very shallow. The fragment O_ε2_(Glu222)-H_W2_-Wat^†^ is flexible, showing some features of the H_3_O^+^ species attached to Glu222 and Tyr203. The descent from the CT/S_0_ point may lead to the hydrated chromophore, back-protonated Tyr203, and deprotonated Glu222.

## 3. Models and Methods

Ref. [14] describes the computational approaches used to construct a full-protein model of the ON-state of Dreiklang by quantum mechanics/molecular mechanics (QM/MM) methods, starting from the coordinates of heavy atoms of the crystal structure PDB ID 3ST2 [1]. Relevant technical details of this construct are summarized in the Supporting Materials for the present paper. As shown in the panels in Figure 1, the constructed model comprises the chromophore in the *cis*-neutral form, and the side chains of His145 (protonated at N_ε_) and Glu222 in the neutral form. This choice matches best [14] the observed absorption spectra of the protein [1]. This model serves as an initial template of the full-protein structure in the ON-state for the simulations described in this work. Optimization of the geometry parameters in the ground electronic state and calculation of energies were performed with the NWChem software package [18]. The density functional theory M06-L functional [19] (as in Refs. [13,14]) and the 6-31G * basis set were used to describe the 155-atomic QM subsystem composed of molecular groups of Chro, Gln94, Arg96, His145, Tyr203, Ser205, Glu222, and water molecules, whereas the AMBER99 force field parameters [20] were applied to describe the MM subsystem.

Scans on the excited-state PES, a search of the conical intersection points, and calculations of the vertical excitation energies and oscillator strengths were carried out for a large molecular cluster, which was almost identical to the QM subsystem in QM/MM simulations in the full-protein model. Figure 2 shows this cluster in the configuration of the ON-state. All calculations for cluster models were performed with the Firefly package [21]. The state-averaged CASSCF method with the distribution of 8 electrons over 6 complete active orbitals represented by the 6-31G basis set (SA2-CASSCF(8/6)/6-31G) was used to locate structures on the excited-state PES and the minimum energy conical intersection points. The use of larger active spaces and larger basis sets in SA-CASSCF calculations of energy derivatives for the 158-atomic cluster is prohibitively expensive. The carbon atoms of all selected amino acid side chains at the border of the cluster with the remaining part of the protein were fixed at the positions optimized in QM/MM calculations. Calculations of the vertical excitation energies and oscillator strengths of electronic transitions were carried out with the algorithms of the extended multiconfigurational quasi-degenerate perturbation theory in the second order (XMCQDPT2) [22] based on the SA-CASSCF(16/12)/cc-pVDZ wavefunctions, as in Ref. [14].

Structures near the potential barriers were optimized in the constrained minimizations and verified by computing energy profiles in the forward and backward directions along reaction coordinates.

## 4. Discussion

The first attempt to dissect the reaction mechanism of the light-induced chromophore hydration in Dreiklang was described by Lacombat et al. [12] following their femtosecond spectroscopy experiments. The authors of a later study [14] formulated critical comments on this tentatively proposed mechanism and suggested an alternative scheme via the results of computational simulations. The key finding of Ref. [14] is that the population of the excited-state level of a charge transfer character in the ON-state, which is nearly isoenergetic with the locally excited state, is an essential step in the ON → OFF photochemical reaction in Dreiklang. The preliminary characterization of the reaction intermediates described in Ref. [14] is based on AIMD molecular dynamics simulations carried out with the Q-Chem program [27,28]. The ONIOM version [29] of the QM(DFT(ωB97X-D))/MM(CHARMM27) approach was applied to analyze 250 fs trajectories for the two lowest electronic states obtained in the TD DFT approach. This analysis suggested several possible candidates for the reaction intermediate X, albeit lacking a confirmation of the observed [12] transient absorption band of X and reliable estimates of its energy in the ground electronic state.

In the present work, we used complementary simulation tools as compared to Ref. [14] to characterize the evolution of the system in the excited state. Namely, a combination of the cluster model and the QM/MM technique allowed us to explore the relief of the excited-state PES on the charge transfer character, to find the relevant conical intersection points, and to trace the evolution of the system on the way to the reaction product. Thus, the simulations described in the present work allow us to successfully complete the theoretical analysis and characterize the chain of transformations in Dreiklang after the illumination of its ON-state. In particular, the nature of the critical reaction intermediate X is established, and the proton transfer routes in the chromophore-containing pocket are indicated. Although free energy calculations are preferable to furnish reaction profiles, the QM/MM-based energy diagram shown in Figure 6 well clarifies the reaction route.

It is worth noting that the importance of the topic of chemical reactions with chromophores in fluorescent proteins extends beyond consideration of Dreiklang only. The variant Dreiklang was engineered from the *Aequorea victoria* GFP by introducing a series of replacements of amino acid residues in the protein. Recently, several new natural fluorescent proteins from *Aequorea* species have been discovered [16]. According to the authors’ suggestion, one of these proteins, AausFP4, with the chromophore matured from the Ala65-Tyr66-Gly67 tripeptide, shows photochromic behavior similar to Dreiklang. Specifically, when expressed and/or stored in the dark, AausFP4 reaches an equilibrium state with a major absorbance peak at 338 nm, indicating that the chromophore is neutral, but missing at least one double bond relative to a mature GFP-type chromophore. With exposure to UV light, AausFP4 fully converts to an anionic GFP-like state with 477-nm peak absorbance. This transformation is reversible by exposure to bright blue light or by storage in the dark, as in Dreiklang. Another interesting development was recently reported by Sugiura and Nagai [17]. The authors engineered a novel bright fluorescent protein, Sumire, with the shortest wavelength (414 nm) of any fluorescent protein reported to date. Motivated by Dreiklang’s properties, the authors assigned the violet fluorescence to a hydrated GFP-like chromophore analogous to that in the OFF-state in Dreiklang.

## 5. Conclusions

This study reports the following new results for the photochemistry of the fluorescent protein Dreiklang, the photoswitchable properties of which are regulated by the hydration/dehydration reaction of its GFP-like chromophore. Following the pathway from the charge transfer excited-state level, we located the minimum energy conical intersection point, the descent from which to the ground-state potential energy surface allowed us to firmly characterize the intermediate photoproduct in the ground state and the route to the reaction product. The intermediate, termed X, was observed in femtosecond studies but unjustly assigned to an adduct of the anionic chromophore and the water molecule [12]. Consistently with the preliminary characterization of X in Ref. [14], its nature corresponds to the complex of the cationic chromophore and the anionic Tyr203 residue and the water molecule. We almost precisely reproduce the experimental absorption bands in Dreiklang, including the transient absorption band in X at 450 nm observed in femtosecond experiments, and demonstrate that a low-energy barrier separates this intermediate from the reaction product. Unlike previous proposals [12,14], we show here that the side chain of Glu222 does not serve as a proton shuttle in the hydration reaction; correspondingly, only the proton and the hydroxyl from the reactive water molecule occur in the hydrated chromophore.

## Figures and Tables

**Table 1:** Comparison of the computed S_0_ → S_1_ excitation bands and experimental absorption bands. Structure ON-A refers to the protein in the ON-state with the neutral Chro (Figure 3b); structure ON-B refers to the ON-state with the anionic Chro (Figure 3c).

Structure	Excitation Energy, eV	Wavelength, nm	Oscillator Strength	Experimental Data [1,12], eV/nm
ON-A	2.80	442	0.30	3.01/411–413
ON-B	2.46	503	0.78	2.43/511
X	2.79	444	0.20	2.76/450
OFF	3.57	347	0.46	3.65/340

## Data Availability

The files with atomic coordinates of the stationary points on potential energy surfaces in the pdb-format are deposited to the general-purpose open-access repository ZENODO, which can be accessed via https://doi.org/10.5281/zenodo.7323258, accessed on 4 January 2023.

## Supplementary Materials

The following supporting information can be downloaded at: https://www.mdpi.com/article/10.3390/molecules28020505/s1, Figure S1: Tentative transformations in the chromophore-containing pocket. The figure is drawn by motifs of Scheme 2B in Ref. [12]; Figure S2: Tentative transformations in the chromo-phore-containing pocket. The figure is drawn by motifs of Figure 13 in Ref. [14]; Sections S1, S2: Illustration of the earlier proposed mechanisms; Section S3: Details of the construction of the full-protein model; Section S4: Description of the files with atomic coordinates in the pdb-format deposited to the general-purpose open-access repository ZENODO in Refs. [30,31], which can be accessed via https://doi.org/10.5281/zenodo.7323258, accessed on 15 November 2022.
