# Supplementary material for: Modeling Light-Induced Chromophore Hydration in the Reversibly Photoswitchable Fluorescent Protein Dreiklang
Source: Molecules. 2023 Jan 4;28(2):505. doi: 10.3390/molecules28020505 (PMC9863226; doi:10.3390/molecules28020505)
Supplement: Supplementary file 1 [file molecules-28-00505-s001.zip › molecules-2081595-supplementary.pdf]

Supporting materials

for

Modeling Light-Induced Chromophore Hydration in the Reversibly Photoswitchable Fluorescent Protein Dreiklang

Bella L. Grigorenko [1,2], Igor V. Polyakov [1,2] and Alexander V. Nemukhin [1,2,*]

[1] Department of Chemistry, M.V. Lomonosov Moscow State University, Moscow, 119991, Russia

[2] N.M. Emanuel Institute of Biochemical Physics, Russian Academy of Sciences, Moscow, 119334, Russia

* Corresponding author:

Prof. Alexander V. Nemukhin, Chemistry Department, Lomonosov Moscow State University, Leninskie Gory 1/3, Moscow, 119991, Russian Federation

E-mail: anemukhin@yahoo.com; anem@lcc.chem.msu.ru

Section S1. Illustration of the mechanism of the light-induced chromophore hydration reaction in Dreiklang proposed in Ref. [S1].

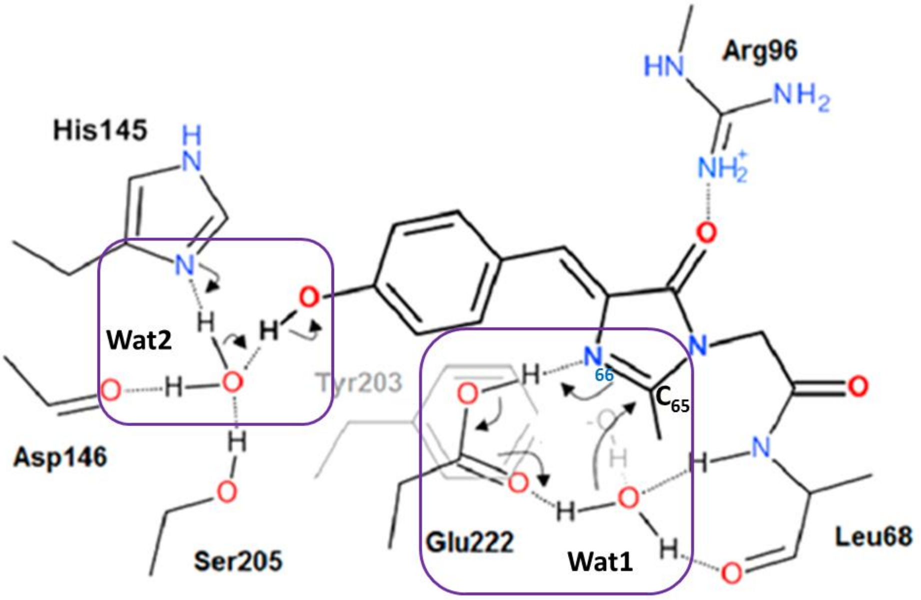

Figure S1. Tentative transformations in the chromophore-containing pocket. The figure is drawn by motifs of Scheme 2B in Ref. [S1].

Figure S1 shows chemical transformations in the chromophore-containing pocket assumed in Ref. [S1]. Two magenta frames distinguish the critical areas. Two water moleculesWat1 and Wat2 participate in the reaction. At the first step (see the left area), Wat2 assists in the proton transfer from the P-ring of the chromophore to create the protonated His145 chain. The emerged anionic chromophore stimulates protonation of the I-ring at the N_{66} atom due to the proton transfer from Glu222 (see the right area). Then the reactive water molecule Wat1 splits providing its proton to Glu222 (restoring the neutral form of Glu222) and the hydroxyl anion to C_{65}.

Section S2. Illustration of the mechanism of the light-induced chromophore hydration reaction in Dreiklang proposed in Ref. [S2].

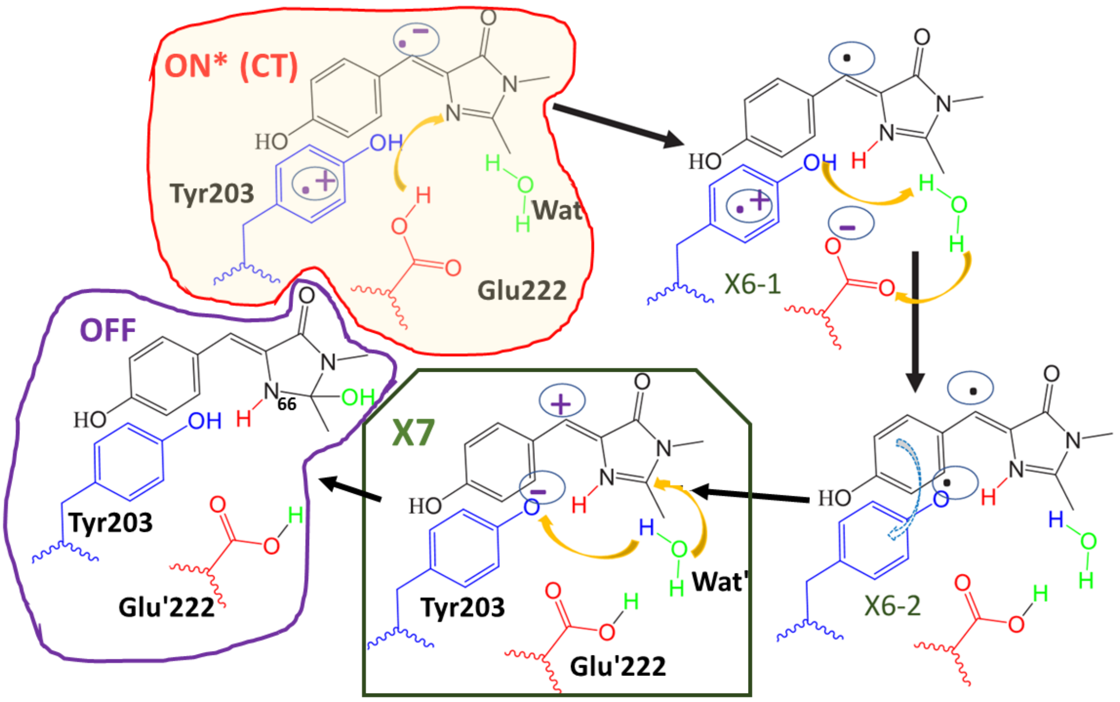

Figure S2. Tentative transformations in the chromophore-containing pocket. The figure is drawn by motifs of Fig. 13 in Ref. [S2].

The upper left panel (ON* (CT)) indicates that the excitation to the charge-transfer level in the ON-state initiates the process. Proton transfer events (from Glu222 to the N_{66} atom of Chro and long the chain Tyr203-Wat-Glu222) might lead to possible reaction intermediates X6-1 and X6-2 and a transient water molecule Wat'. Subsequent electron transfer leads to the intermediate X7 with the ion pair $Chro^{+}$-$Tyr203^{-}$. Re-protonation of Tyr203 and binding of the hydroxyl to the C_{65} atom completes the reaction.

Section S3. Preparation of the full-protein model system in the ON-state.

To construct model molecular systems, we started from the coordinates of heavy atoms in the crystal structure PDB ID 3ST21 (chain A) from the Protein Data Bank. This is the equilibrium structure of Dreiklang, from which the OFF- and ON-states are derived experimentally, as characterized by the crystallography studies [S3].We added hydrogen atoms using molecular mechanics tools, we assumed that side chains of Arg and Lys are positively charged and that side chains of Glu and Asp are negatively charged. The protein was fully surrounded by explicit water molecules. The system with the original (non-hydrated) chromophore was equilibrated in preliminary molecular dynamics simulations with the CHARMM36 force field [S4] and parameters for the chromophore taken from Ref. [S5]. In QM/MM optimization, a large fraction of the chromophore-containing pocket was assigned to the QM-part. Specifically, atoms of Chro, side chains of Gln94, Arg96, His145, Tyr203, Ser205, Glu222, and 7 water molecules were included in QM.

References

(S1) Lacombat, F.; Plaza, P.; Plamont, M.-A.; Espagne, A. Photoinduced Chromophore Hydration in the Fluorescent Protein Dreiklang Is Triggered by Ultrafast Excited-State Proton Transfer Coupled to a Low-Frequency Vibration. *J. Phys. Chem. Lett.* **2017**, *8*, 1489-495.

(S2) Sen, T.; Ma, Y.; Polyakov, I.V.; Grigorenko, B.L.; Nemukhin, A.V.; Krylov, A.I. Interplay between Locally Excited and Charge Transfer States Governs the Photoswitching Mechanism in the Fluorescent Protein Dreiklang. *J. Phys. Chem.* **2021**,125, 757-770.

(S3) Brakemann, T.; Stiel, A.C.; Weber, G.; Andresen, M.; Testa, I.; Grotjohann, T.; Leutenegger, M.; Plessmann, U.; Urlaub, H.; Eggeling, C.; et al. A Reversibly Photoswitchable GFP-Like Protein with Fluorescence Excitation Decoupled from Switching. *Nat. Biotechnol.* **2011**, *29*, 942−947.

(S4) Best, R.B.; Zhu, X.; Shim, J.; Lopes, P.E.M.; Mittal, J.; Feig, M.; MacKerell, A.D. *J. Chem. Theor. Comput.* **2012**, *8*, 3257–3273.

(S5) Reuter, N.; Lin, H.; Thiel, W. Green Fluorescent Proteins: Empirical Force Field for the Neutral and Deprotonated Forms of the Chromophore. Molecular Dynamics Simulations of the Wild Type and S65T Mutant. *J. Phys. Chem. B* **2002**, *106* (24), 6310–6321.

Section S4. Description of the files with the structures, which are deposited to the general-purpose open-access repository ZENODO (can be accessed via https://doi.org/10.5281/zenodo.7323258).

Files with the atom coordinates (pdb-format) computed in QM/MM calculations:

QMMM_ON_neutralChro_minimum.pdb – the minimum-energy structure of the ON-state with the neutral chromophore (Fig. 3b in the manuscript)

QMMM_ON_anionicChro_minimum.pdb - the minimum-energy structure of the ON-state with the anionic chromophore (Fig. 3c in the manuscript)

QMMM_X_minimum.pdb – the minimum-energy structure of the reaction intermediate X (inset in Fig. 6 in the manuscript)

QMMM_OFF_minimum.pdb - the minimum-energy structure of the reaction product (Fig. 5a in the manuscript)

Files with the atom coordinates (pdb-format) computed in SA-CASSCF calculations:

CAS_ON_minimum_S0.pdb - the minimum-energy structure of the ON-state with the neutral chromophore (Fig. 4a in the manuscript)

CAS_CT_minimum.pdb - the minimum-energy structure on the charge-transfer excited-state potential energy surface (Fig. 4b in the manuscript)

CAS_CI_protGlu.pdb – the minimum-energy conical intersection point CT/S0 with the protonated form of Glu222 (Fig. 4c and Fig. 7a in the manuscript)

CAS_CI_deprotGlu.pdb – the minimum-energy conical intersection point CT/S0 with the deprotonated form of Glu222 (Fig. 7b in the manuscript)

CAS_X_S0.pdb - the minimum-energy structure of the X reaction intermediate on the ground-state potential energy surface (Fig. 4d in the manuscript)

CAS_OFF_minimum_S0.pdb - the minimum-energy structure of the reaction product (OFF-state) on the ground-state potential energy surface (Fig. 4e in the manuscript)
